# Continuous evaluation of exosomatic electrodermal activity in patients with primary palmoplantar hyperhidrosis

**DOI:** 10.31744/einstein_journal/2024AO1152

**Published:** 2024-11-21

**Authors:** Rafael José Silveira, Carolina Carvalho Jansen Sorbello, Nelson Wolosker, José Ribas Milanez de Campos, João José de Deus Cardoso, Alexandre Sherlley Casimiro Onofre

**Affiliations:** 1 Universidade Federal de Santa Catarina Hospital Universitário Department of Surgical Clinic Florianópolis SC Brazil Department of Surgical Clinic, Hospital Universitário, Universidade Federal de Santa Catarina, Florianópolis, SC, Brazil.; 2 Hospital Israelita Albert Einstein São Paulo SP Brazil Hospital Israelita Albert Einstein, São Paulo, SP, Brazil.; 3 Universidade de São Paulo Faculdade de Medicina Department of Vascular and Endovascular Surgery São Paulo SP Brazil Department of Vascular and Endovascular Surgery, Faculdade de Medicina, Universidade de São Paulo, São Paulo, SP, Brazil.; 4 Hospital Israelita Albert Einstein Faculdade Israelita de Ciências da Saúde Albert Einstein São Paulo SP Brazil Faculdade Israelita de Ciências da Saúde Albert Einstein, Hospital Israelita Albert Einstein, São Paulo, SP, Brazil.; 5 Universidade de São Paulo Faculdade de Medicina Department of Thoracic Surgery São Paulo SP Brazil Department of Thoracic Surgery, Faculdade de Medicina, Universidade de São Paulo, São Paulo, SP, Brazil.; 6 Universidade Federal de Santa Catarina Department of Clinical Analysis Florianópolis SC Brazil Department of Clinical Analysis, Universidade Federal de Santa Catarina, Florianópolis, SC, Brazil.

**Keywords:** Hyperhidrosis, Palmoplantar sweating, Sweat, Anxiety, Depression, Perception, Galvanic skin response, Surveys and questionnaires

## Abstract

**Objective:**

To objectively evaluate sweat intensity in patients with primary hyperhidrosis by measuring electrodermal activity using a continuous exosomatic technique without external stimuli.

**Methods:**

This prospective and transversal study analyzed 10 patients with palmoplantar hyperhidrosis (HH Group) and 10 individuals without it (Control Group) between January and August 2023. All participants underwent one clinical evaluation and completed two multiple-test questionnaires without any intervention from the interviewer, based on their own estimates of sweat perception and anxiety or depression symptoms. Then, the electrodermal activity was measured with the MP36R, a portable instrument from Biopac Systems Inc. (USA), using exosomatic and direct current techniques. Data were tested for normality using the Shapiro-Wilk test. Descriptive statistics were expressed as medians and interquartile ranges (p_25-75_) for numerical variables and as absolute and relative frequencies for categorical variables. Fisher's exact test and Mann-Whitney U test were used to compare the groups. Statistical significance was set at p<0.05.

**Results:**

Palmoplantar sweating was significantly higher in the HH Group than in the Control Group (p<0.01). There were no statistically significant differences in anxiety and depression levels between the groups (p=0.87; p=0.32). Continuous exosomatic electrodermal activity resulted in significantly higher skin conductance in patients with hyperhidrosis than in controls.

**Conclusion:**

Continuous exosomatic electrodermal activity without external stimuli proved to be suitable for the objective analysis of patients with hyperhidrosis in a non-invasive and sensitive manner.

## In Brief

This study investigated variations in the electrodermal activity of the skin, measured by a portable biosensor, in patients with palmoplantar hyperhidrosis, using the exosomatic technique, direct electric current, and no external stimulus. The technique proved to be suitable, with a clinical correlation, for objectively analyzing patients with hyperhidrosis in a sensible non-invasive way.

## Highlights

■ Continuous exosomatic electrodermal activity was associated with higher skin conductance levels in patients with hyperhidrosis.
■ Palmoplantar sweating was significantly higher in the hyperhidrosis group than in the control group (p<0.01).
■ Electrodermal activity is an effective and simple method for objectively quantifying sweating in patients with hyperhidrosis.

## Graphical abstract

**Figure:**
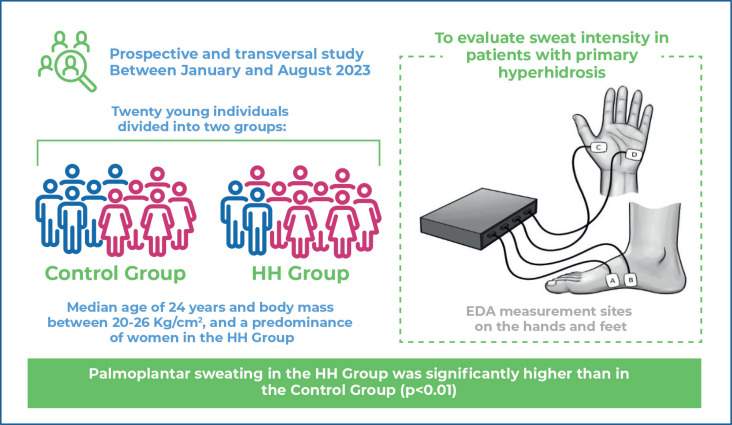

## INTRODUCTION

Primary hyperhidrosis (HH) is a chronic disease of unknown etiology. It is suspected to be caused secondary to the hyperactivity of the sympathetic nervous system (SNS), creating excessive sweating unrelated to the maintenance of body temperature.^(1)^ Although it affects both sexes,^(2)^ regardless of race, color, or social class, women affected by the disease tend to seek medical help more often than their male counterparts.^(2,3)^ In addition, HH significantly impairs patients’ quality of life (QoL).^(3,4)^

The diagnosis is primarily clinical; however, complementary tests may be necessary if secondary hyperhidrosis is suspected. HH can be treated clinically with anticholinergics, such as oxybutynin hydrochloride, or surgically with video-assisted thoracic sympathectomy, which is the only definitive treatment.^(4,5)^

The degree of sweating and its impact on the QoL of patients with HH is often evaluated using specific questionnaires.^(6)^ This method is considered to be the gold standard because it is inexpensive, simple, and can be easily replicated on a large scale. Despite these qualities, questionnaires depend on good translations and are subjective, because they are based on personal interpretation.^(7,8)^ However, reliable and easily reproducible objective methods for diagnosing and stratifying hyperhidrosis are yet to be standardized.

Patients with HH can be objectively analyzed using physical tests such as pad gloves or techniques that measure transepidermal sweat loss.^(9)^ However, these methods, despite their merits, are rarely used in medical practice due to difficulties in interpretation and impracticality in clinical situations. This emphasizes the need for reliable and practical diagnostic tools.

One novel method described in the literature for assessing HH is electrodermal activity (EDA) measurement, which is widely used to analyze autonomic responses in psychophysiological studies.^(10)^ Electrodermal activity is often used to evaluate responses to fear, anxiety, trauma, and stress, among others.^(11)^ The method assesses electrical changes in the skin resulting from sweat activity. An increase in the hydroelectrolytic concentration in sweat glands reduces the skin's electrical resistance, causing an inversely proportional increase in skin conductance (SC), the main electrophysiological measure of this phenomenon.^(12)^

Eectrodermal activity can be measured using the endosomatic technique, which does not apply an external electric current, or the exosomatic technique, which applies an external electric current. Some studies have analyzed patients with HH using an endosomatic technique, whereas others have used an exosomatic technique associated with external stimulation. However, the results are inconsistent.^(13)^

In 2008, Tronstad et al.^(14)^ developed a portable device to measure EDA in a simple manner. The device is linked to a computer system that allows for continuous exosomatic analysis without external stimuli.

## OBJECTIVE

This study aimed to prospectively analyze the continuous application of the exosomatic technique, without an external stimuli, with a portable device to measure electrodermal activity in individuals with and without palmoplantar hyperhidrosis.

## METHODS

### Study Design

This prospective cross-sectional study followed the guidelines of the local Human Research Ethics Committee and was approved by the Ethics Committee of *Universidade Federal de Santa Catarina* (UFSC) (CAAE: 43287321.8.0000.0121; #4.712.363). The patients were invited to participate in the study and provided informed consent.

### Settings

Between January and August 2023, we measured the intensity of sweating in 10 patients clinically diagnosed with palmoplantar HH (HH Group) and 10 individuals without it (Control Group), using the portable device mentioned above. The clinical diagnosis was based on the Hornberger criteria^(15)^ using the frequency of sweating, laterality of symptoms, impact on routine activities, family history of HH, age, and reduction in sweating during sleep.

All the participants were evaluated once. During the visit, patients underwent a thorough clinical assessment and completed two questionnaires (HDSS and HADS), without any intervention or advice from the interviewer, based solely on their own estimates. Finally, they were subjected to continuous exosomatic EDA examination.

### Measurements

The intensity of sweating was measured using the Hyperhidrosis Disease Severity Scale (HDSS), which consists of a questionnaire comprising one simple and direct question referring to the patient's degree of tolerance to the symptoms of HH and the interference of sweating in their daily lives. The scores range from 1 to 4, with 1 indicating no noticeable sweating and no interference in day-to-day life; 2, tolerable sweating with little interference; 3, little tolerable sweating with frequent interference; and 4, intolerable sweating with constant interference.^(7)^

Anxiety and depression levels were estimated using the Hospital Anxiety and Depression Scale (HADS), which comprises two subscales with seven questions each about anxiety and depression. These questions have four levels of classifications between 0 and 3. A score of 0-7 suggests the absence of anxiety and depression symptoms; 8-15, possible case of anxiety or depression; and 16-21, probable case of anxiety or depression.^(16)^

We measured EDA using an MP36R, a portable, noninvasive instrument from Biopac Systems Inc., USA, following the Psychophysiology American Society guidelines.^(17)^ The chosen technique was exosomatic with constant electric flow of 0.5 Volts, sampling rate of 200,000 Hz (Hertz), high pass filter off, low pass filters at 66.5 Hz and 38.5 Hz, with disposable electrodes of silver hydrochloride and without any external stimuli. The electrophysiological measurements were amplified and sent to the computer via a USB cable for interpretation using AcqKnowledge software.

**Figure 1:**
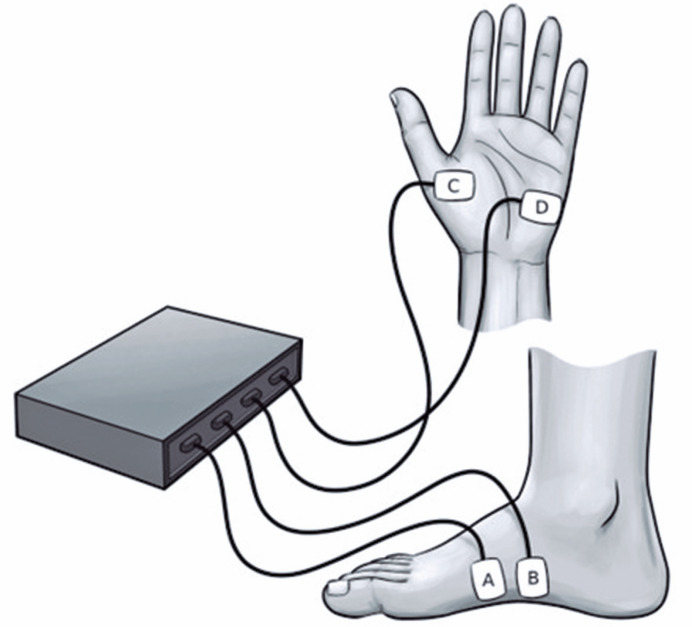
Electrodermal activity measurement sites on the hands and feet

The parameters were initially collected on the hands (right and left) in the thenar and hypothenar regions, because they are more closely related to thermoregulatory function^(12)^ while the phalanges are associated with emotional factors. Next, the EDA on the feet was measured on both sides (right and left, respectively), on the medial surface of the plantar arch (Figure 1), as this region has a higher concentration of sweat glands and is free of possible confounding factors, such as dry skin on the soles of the feet or the presence of calluses.

The experiment was conducted exclusively by the researcher in an air-conditioned room with a temperature between 21°C and 23°C and humidity of 60% to 65%, with approximately 12m^2^ and artificial light, without any external stimuli. All measurements were obtained once with the participant sitting comfortably after 10 min of rest.

Electrodermal activity levels were estimated by measuring the skin conductance level (SCL) and skin conductance area (SCA), and measurements were taken for 5 uninterrupted minutes in each dermatome. The SCL directly measures skin EDA, which is expressed in microsiemens. The SCA was obtained by calculating the area under the curve in the SCL *versus* time graph.

### Statistical analysis

First, anthropometric and demographic data were analyzed, followed by the intensity of HH, level of medium EDA, and EDA by area.

All the data was stored in a Microsoft Excel® spreadsheet and then imported into the IBM Statistical Package for the Social Sciences version 23 (International Business Machines, NY, USA) program for analysis. The normality of the data was examined using the Shapiro-Wilk test. Descriptive statistics were expressed as measures of the central tendency and dispersion of numerical variables, while categorical variables were expressed as absolute and relative frequencies. Fisher's exact test, Student's *t*-test (for independent samples), and Mann-Whitney U test were used to compare the groups. Statistical significance was set at a threshold of p<0.05.

## RESULTS

The sample consisted of young individuals, with a median age of 24 years and body mass index (BMI) between 20-26Kg/cm^2^, and a predominance of women in the HH group. The patients did not report HH at any sites other than the palmoplantar region. The distribution of participants and their clinical characteristics are shown in table 1.

**Table 1:** Participants distribution and clinical characteristics according to their groups

Variables		Group		p value *
		Control	HH	
Sex				0.16
	Male	5 (50.0)	2 (20.0)	
	Female	5 (50.0)	8 (80.0)	
Age (MD; p_25-75_)		26.0 (24.7–26.2)	22.0 (18.7–23.2)	0.04
BMI (MD; p_25-75_)		22.0 (21.4–25.1)	21.3 (18.3–26.6)	0.40
HDSS				
	Hand	1.1	3.8	<0.01
	Foot	1.1	3.4	<0.01
HADS				
	Anxiety	9.2 (±4.6)	9.5 (±4.4)	0.87
	Depression	4.7 (±2.7)	6.4 (±4.7)	0.32

* Fisher's exact test for categorical variables; Mann-Whitney U test for numerical variables.

BMI: body mass index; MD: median; P_25-75_: percentile_25-75_; HDSS: Hyperhidrosis Disease Severity Scale; HADS: Hospital Anxiety and Depression Scale.

The intensities of palmar and plantar sweating were significantly higher in the HH Group than in the Control Group (p<0.01). There were no statistically significant differences in anxiety and depression levels between the groups (p=0.87; p=0.32).

The SCL and SCA measurements are listed in table 2. Skin conductance level analysis showed a statistically significant difference in the hands and feet bilaterally, with higher numbers in the HH Group.

**Table 2:** Electrodermal activity measurements in the groups with and without primary hyperhidrosis

Measurements		Group		p value*
		Control	HH	
Mean skin conductance level				
	Right hand	4.4 (2.0)	14.0 (7.8)	<0.1
	Left hand	5.9 (2.8)	12.8 (7.3)	0.01
	Right foot	2.5 (1.6)	12.7 (9.7)	<0.01
	Left foot	2.2 (1.9)	15.4 (13.2)	0.01
Skin conductance area				
	Right hand	107.1 (53.2–361.4)	356.3 (198,7–467.4)	0.04
	Left hand	265.5 (69.9–390.7)	402.5 (199.2–596.4)	0.18
	Right foot	26.8 (17.0–127.6)	195.6 (111.8–350.2)	<0.01
	Left foot	23.1 (10.8–156)	396.4 (136.1–621.2)	0.02

The results are expressed as median and interquartile range (25th and 75th percentiles).

* p<0.05.

HH: primary hyperhidrosis; uS: microsiemens; uS/cm^2^: microSiemens per square centimeter.

The mean amplitude of skin conductance was significantly higher in the HH Group than in the Control Group in all dermatomes: right hand [t (18) −3.^739^; p<0.01], left hand [t (18) −2.754; p=0.01], right foot [t (18) −3.281; p<0.01], and left foot [t (18) −2.790; p=0.03]. Therefore, it can be inferred that the EDA intensity was higher in the HH Group than in the Control Group (Table 2).

Concerning the EDA measured by SCA, there was no statistically significant difference between the groups on the left hand [U = 28.00, p=0.18]. However, in other regions, there was a significant difference, namely in the right hand [U = 23.00; p=0.04], right foot [U = 15.00; p<0.01], and left foot [U = 11.00; p<0.01]. These findings indicated that the intensity of EDA was higher in the HH group than in the Control Group (Table 2).

## DISCUSSION

HH is a chronic disease with clinical and surgical relevance that can cause significant psychosocial damage.^(18)^ It can be treated with medication^(19,20)^ or surgery.^(21)^ Therefore, the study of etiologies, diagnostic methods, risk factors, affected groups, types of treatment, and clinical and psychological consequences is not only essential for its management but also for defining the appropriate course of action to optimize the patients’ QoL.^(22)^ Our study presents a new technique for objectively measuring sweating intensity in patients with HH and could contribute significantly to this goal.

To assess HH, several questionnaires were used to quantify sweating, analyze QoL, and assess patient satisfaction. However, few methods have objectively quantified sweating.

Transepidermal water loss measurement, widely cited in the literature, consists of evaluating the difference in weight of a compress used to dry the area under analysis (hands, feet, axilla, etc.) to quantify sweat, and thus, classify and monitor patients with HH. Although simple and easy to apply, it depends on an external stimulus (stress) and may be inaccurate because it depends on the absorption capacity of the pad and losses secondary to sweat evaporation. The "pad glove" method is very similar, differing in the use of a glove instead of a compress to absorb and weigh sweat.

Other less widespread methods such as the "VapoMeter" and the "ventilated capsule system" seem to add precision to the measurements because they are connected directly to the sweat-producing areas and have systems to remove artifacts. However, they may increase the assessment cost and are difficult to reproduce on a large scale.

In this study, we presented a novel technique that efficiently analyzes sweating intensity in patients with HH. The technique objectively quantified sweating and revealed significantly higher EDA values in the HH Group than in the Control Group, advancing our understanding of HH.^(9,14,23)^

The sample comprised patients who had sought treatment for HH, were under 30 years of age, had a normal BMI, and were mostly female.^(24)^ The Control Group was carefully selected to match the epidemiological profiles of the patients to ensure the integrity of the results.

The HDSS questionnaire was used to measure the sweating intensity. This questionnaire was previously translated into Portuguese and validated by Varella et al., demonstrating a statistical correlation with other established questionnaires, such as the QoL^(25)^ and SEQ.^(7)^ In addition, the HDSS is easy to reproduce and understand, with adequate sensitivity to better assess patients with HH. To measure patients’ anxiety and depression, we chose the HADS,^(16)^ a tool widely used and validated in various hospital centers as well as in community medical practice, as an aid in measuring psychological distress secondary to multiple pathologies.

Electrodermal activity refers to the electrical phenomena that occur in the skin and reflects activity within the sympathetic axis of the autonomic nervous system. The basic principle for measuring EDA is that water and electrolytes are controlled by individual parts of an organism, with the ability to transmit a weak electric current between two electrodes placed on the surface of the skin.^(26)^ This provides a sensitive and convenient measure to assess changes in sympathetic arousal associated with emotion, cognition, and attention. Thus, EDA represents changes in the electrical properties of the skin, particularly skin resistance, resulting from sweat activity, which alters the hydroelectrolytic concentrations in the glands.^(27)^ Therefore, we hypothesized that the basal sudomotor activity could be assessed based on changes in the electrical properties of the skin and quantified using an electrophysiological stimulus transducer.

Electrodermal activity measurements depend on the density of eccrine sweat glands at the site and the level of sympathetic stimulation in these glands^(28)^ to quantify sweating.^(11,12,29)^ This measurement did not interfere with the parasympathetic system. The palms and soles of the feet have a higher concentration of sweat glands and greater sweat secretion, which favors measuring EDA in patients with palmar-plantar HH.

The main extrinsic and intrinsic factors that can interfere with electrodermal measurements are artifacts, weather conditions at the time of measurement, mental and physical activity levels, and the use of substances or medications that impact the SNS. Therefore, the standardization of EDA measurements in accordance with the guidelines of the American Psychophysiological Society^(17)^ is essential for reliable analyses. Thus, the patients were placed in a noise-free room with a fixed temperature of 21°C to 23°C and asked to rest for 10 minutes before the measurement, which is painless.

In this study, we set a time of 5 minutes for continuous EDA measurement in each dermatome based on the studies by Dooren and Boucein, who recommended a minimum time of 3 minutes for each clinical scenario owing to the intermittent nature of the disease and the variability of the measurements.^(12,28)^

Electrodermal activity changes were assessed both by the mean amplitude of skin conductance and by the area of skin conductance because, according to Boucsein et al.^(12)^ this metric reinforces the importance of time in the measurement.

This study showed significantly higher EDA levels in the HH Group than in the Control Group when measured without external stimuli and under continuous observation. Although the study did not include a specific stress scenario, the levels of anxiety and depression between the groups did not differ, which strengthens the hypothesis that the higher levels of EDA in the HH Group were solely owing to the disease.

Studies analyzing EDA using endosomatic or exosomatic techniques with external stimuli to evaluate HH have shown conflicting results, particularly when comparing EDA before and after sympathectomy.^(13,29)^

Manca et al. showed a greater intensity of EDA in patients with HH than in those without it,^(30)^ using an examination technique with external electrical stimulus. The exosomatic technique in HH was used by Tronstad et al*.* in 2014, who measured the EDA in 22 patients undergoing axillary sweat gland resection and showed a good correlation between electrodermal measurements, clinical assessment, and sweat dosage.^(31)^ However, this study assessed patients with axillary HH and used alternating current (AC) associated with external stimulation. The use of EDA with an exosomatic technique, direct current, and no external stimulus to assess patients with palmoplantar HH was described for the first time in this study.

This study showed no difference in either group's EDA measurements of laterality in the hands or feet. According to Caruelle et al., in a review article on EDA, there are no significant differences in the measurements according to the influence of each cerebral hemisphere.^(32)^

Regarding sex, Bari et al. found no significant differences in the EDA performed at rest or under stress in 30 men and 30 women.^(33)^ Similarly, Quasin et al. found no differences between the sexes in 36 healthy participants aged 21-24 years.^(26)^ Our study results were consistent with these findings.

The main limitations of objective measures for analyzing autonomic diseases, especially HH, are the variability and non-specificity of their parameters, cyclical nature of the disease, and technical difficulties involved in conducting them. EDA is an objective measure that can be used in a simple and continuous manner, overcoming these limitations.

The EDA measurements obtained in this study showed greater sweat intensity in patients with HH than in the Control Group using an accessible and non-invasive method with a portable device. Another advantage of this method is the possibility of analyzing the data both in real time and after it has been stored on the cloud, facilitating the use of this psychophysiological parameter in clinical practice.

The data obtained, when compared with clinical assessment using a questionnaire (HDSS), showed a statistically significant correlation, as EDA levels were considerably higher in the hands and feet of patients with HH than in those without it.

### Study limitations

This study aimed to evaluate the use of the EDA in objective analysis of patients with HH using an exosomatic technique without external stimulation with the aforementioned device, which has not yet been covered in the literature. Therefore, the secondary findings may have been limited owing to the chosen sample.

Due to the limited number of participants, the comparative analysis of the groups based on sex may not be consistent with previous data showing a higher prevalence of women with HH, which may also be related to the fact that women tend to seek medical help more often than men. In addition, as the diagnosis of HH causes significant psychosocial damage to patients, it is related to a higher prevalence of anxiety and depressive symptoms, which was not confirmed in this study. This may also be due to the limited number of participants.

However, the correlation between the clinical and EDA measurements in the assessment of excessive sweating in the study participants reinforces that the central objective was met by the chosen sample.

## CONCLUSION

Electrodermal activity measured using the continuous exosomatic technique without external stimuli can be an objective and accurate measure of sweat gland activity and sweat intensity in patients with hyperhidrosis, with a clinical correlation. Thus, the technique in this study may represent a feasible and reliable tool in clinical practice for diagnosing and classifying the severity of hyperhidrosis.
